# *Candida albicans* colonization of the gastrointestinal tract: A double-edged sword

**DOI:** 10.1371/journal.ppat.1009710

**Published:** 2021-07-22

**Authors:** Rebeca Alonso-Monge, Mark S. Gresnigt, Elvira Román, Bernhard Hube, Jesús Pla

**Affiliations:** 1 Departamento de Microbiología y Parasitología-IRYCIS, Facultad de Farmacia, Universidad Complutense de Madrid, Madrid, Spain; 2 Junior Research Group Adaptive Pathogenicity Strategies, Leibniz Institute for Natural Product Research and Infection Biology-Hans-Knoell-Institute, Jena, Germany; 3 Department of Microbial Pathogenicity Mechanisms, Leibniz Institute for Natural Product Research and Infection Biology-Hans-Knoell-Institute, Jena, Germany; 4 Institute of Microbiology, Friedrich Schiller University, Jena, Germany; Stanford University, UNITED STATES

## Summary

*Candida albicans* is not only a common commensal of the vaginal and gastrointestinal tract (GIT) of humans, but also an important cause of infections worldwide and is therefore considered an opportunistic pathogen. *C*. *albicans* can cause superficial but also more severe, frequently life-threatening, systemic infections. The latter may occur when the microbiota is disturbed and immune defenses are compromised, thus allowing the dissemination of the fungus from commensal pools, in particular the GIT, to vital organs. Therefore, gastrointestinal *C*. *albicans* colonization can be seen as a predisposing factor of life-threatening infections. However, recent evidence indicates that commensal coexistence of *C*. *albicans* with the human host is not only detrimental. In fact, beneficial effects of *C*. *albicans* colonization to human health, most likely, have been an evolutionary pressure for its establishment as a commensal. Here, we review recent studies that demonstrate both beneficial and detrimental effects of this pathobiont to human health upon colonization of the human gut.

## Is the gut a source for *C*. *albicans* systemic infections?

Different studies suggested that the gut is the main reservoir from which *C. albicans* can translocate through the intestinal barrier causing blood stream infections (BSIs) [1]. Immunocompromised animals or those with disrupted intestinal barriers can develop BSI leading to dissemination, colonization of vital organs, and death. Molecular typing studies have demonstrated that systemic infections originate from the gastrointestinal tract (GIT) [2]. Genetic similarity between *C*. *albicans* blood isolates and the corresponding strains isolated from stools was found in systemically infected patients [3]. A comparative analysis of the mycobiota in fecal and blood samples from allogenic hematopoietic cell transplant patients has revealed an expansion of the intestinal *Candida* species prior to dissemination [4]. In fact, the majority of intensive care unit (ICU) patients, a major risk group for systemic *Candida* infections, seem to show increased sizes of certain *Candida* populations in the gut [5]. Therefore, microbial dysbiosis and *Candida* overgrowth in the gut can be considered a risk and a source for systemic candidiasis, besides catheter-derived nosocomial candidemia.

## Is intestinal *C*. *albicans* colonization responsible for specific human pathologies?

**Fig 1:**
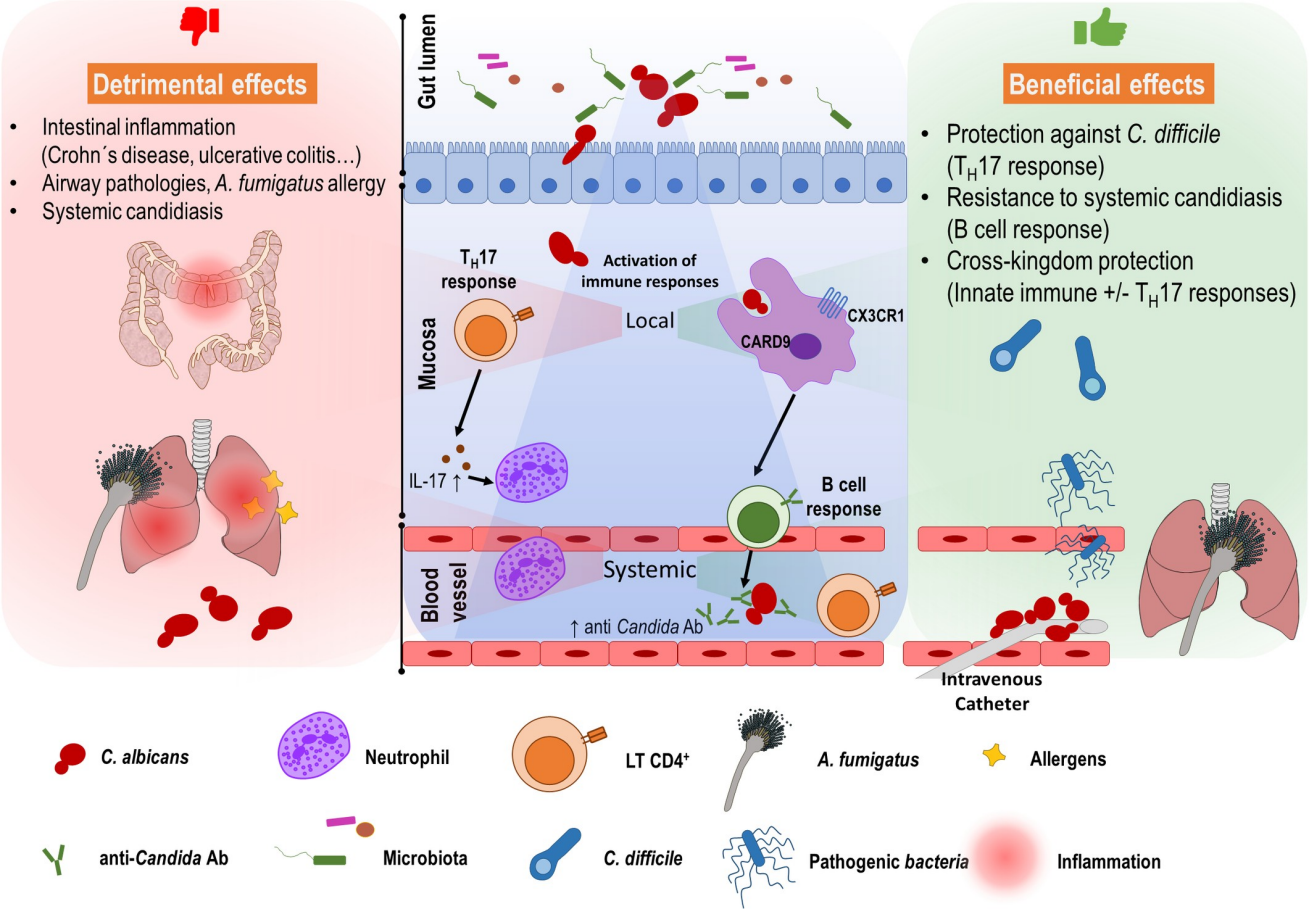
A general scheme of beneficial and detrimental effects of *Candida albicans* colonization in the gut. *C*. *albicans* inhabits the intestinal tract with other fungal and bacterial microbiota, preferably as a yeast form, in healthy individuals. Alteration of this equilibrium (caused by immunological defects, use of broad-spectrum antibiotics, barrier damage, etc.) can lead to dysbiosis. *C*. *albicans* can then translocate from the gut lumen and invade the intestinal mucosa, getting access to blood vessels and causing systemic infections. The presence of *C*. *albicans* as a harmless intestinal commensal, however, induces T_H_17 mediated responses. Neutrophils are attracted to the intestinal mucosa playing a protective role against other pathogens such as *Clostridioides difficile*. This T_H_17 response can exacerbate inflammation in IBD patients or aggravate allergic symptoms to *Aspergillus fumigatus*. CX3CR1+ CARD9+ macrophages mediate induction of B cell responses that generate anti-*Candida* antibodies that protect the host from other fungal systemic infections. A protection against potentially pathogenic fungi or bacteria such as *Staphylococcus aureus* or *Acinetobacter baumanii* has also been reported, indicating cross-kingdom protection. Ab, antibody; IBD, inflammatory bowel disease; IL-17, interleukin 17.

Since most of the human population is colonized by *C*. *albicans*, a colonization of the gut per se can be considered as normal. However, in certain cases, *C*. *albicans* colonization has been associated with intestinal pathologies (Fig 1). Crohn disease (CD), an inflammatory bowel disease (IBD), has long been suspected to arise from inappropriate immune responses to the intestinal microbiota, and compelling evidence indicates that the mycobiota plays an essential role in its etiology. CD patients have elevated antibodies against fungal cell wall sugars (originally called anti-*Saccharomyces cerevisiae* or ASCA antibodies) that can recognize *C*. *albicans*. Additionally, *C*. *albicans* is more frequently isolated from stools of CD patients (see [6] for a review). Intestinal CX3CR1+ monocytes mediate specific antifungal responses, and a CX3CR1 polymorphism (T280M) is associated with reduced ASCA levels and impaired immunoglobulin G (IgG) responses against commensal fungi in humans [7]. Also, ulcerative colitis patients colonized with *C. albicans* experience delayed recovery, and both antifungal therapy and probiotic treatment ameliorate their symptoms [8].

Recent evidence suggests that intestinal *C*. *albicans* colonization can influence certain nonintestinal pathologies. For example, *C*. *albicans* is a potent inducer of T_H_17 responses, which may be beneficial in the gut, but this response also causes the expansion of CD4+ T cells cross-reactive to *Aspergillus fumigatus* antigens [9] or house dust mite extract [10] causing immunopathology in the lung [9]. Interestingly, CD patients have increased numbers of these cells that are also increased in patients with asthma, chronic obstructive pulmonary disease, and cystic fibrosis. The interaction of intestinal immune cells with *C*. *albicans* can exacerbate pulmonary inflammatory responses against airborne fungi or allergens and is believed to contribute to the pathology of certain allergic airway diseases. Different studies propose a connection between *C*. *albicans* colonization and other pathologies, but whether fungal dysbiosis is a consequence or a cause of these pathologies remains to be determined.

## Does *C*. *albicans* gut colonization have beneficial effects for the host?

Although the correlation between *C*. *albicans* gut colonization and disseminated candidiasis has long been known, potential advantages of harboring this fungus as a commensal in the mycobiome has been discussed more recently (Fig 1). For example, *C*. *albicans* colonization has been shown to have a beneficial effect in *Clostridioides difficile* infection [11]. Colonization of *C*. *albicans* may also contribute to protective effects against blood-borne microbial infections as it drives the expansion of T_H_17 CD4+ cells that stimulate the responsiveness of circulating neutrophils [10]. Mice colonized with *C*. *albicans* demonstrated increased resistance against intravenous challenge with *C*. *albicans* [12]. This occurs via innate immune mechanisms that, interestingly, also increase resistance against other fungal (*A*. *fumigatus*) and bacterial (*Staphylococcus aureus* and *Pseudomonas aeruginosa*) infections [13]. Fungal gastrointestinal colonization (the mycobiota) has recently been shown to induce antifungal IgG responses, protecting mice against infection with *C*. *albicans* or *Candida auris* [14]. Therefore, the commensal *C*. *albicans* pool seems to play a role in generating protective immunity against *C*. *albicans* infections, but also other pathogens. Certain *C*. *albicans* antigens such as, for example, Hyr1, promote cross-kingdom protection against *Acinetobacter baumanii* infections [15] reinforcing the idea of benefits underlying gut colonization.

## Is there a preferred morphology associated with gut colonization?

*C*. *albicans* grows as one of two main morphologies, yeast or hyphae. A specific type of cells (called GUT) has been generated in vivo in the mouse gut under specific conditions [16] and is proposed as a commensalism-specific morphotype.

The hyphal morphology is well known to be more associated with host cell invasion and damage. Although yeast cells are more frequently found in the mouse intestine compared to hyphae, a recent study suggests the existence of both morphologies in the gut [17]. However, several studies have shown that the hyphal form seems to be detrimental for gut colonization. For example, experimental evolution selected mutants with hyphal defects after several rounds of gut colonization [13]. Furthermore, transcription factors that promote *C*. *albicans* persistence in gnotobiotic mice regulate yeast versus hyphal formation [18]. Genetically engineered strains either locked in the hyphal form or producing hypha in vivo show reduced intestinal colonization [19,20], and Ume6, a positive regulator of filamentation, inhibits colonization [17]. All these data suggest that hyphal formation does not support gut colonization. However, since both morphologies are found in the gut and most clinical isolates have the potential to produce hyphae, there must be at least a transient selective advantage for hyphal production. Mechanisms underlying these processes could implicate microbiota and/or host responses in the control of fungal morphology. The existence of other niches within the host (e.g., oral cavity) where evolution may have a different impact on *C*. *albicans* morphology [21] could also partially explain these observations.

## Is a *Candida* vaccine needed?

Patients with *Candida* infections due to an impaired immune response or inefficient recognition of *Candida* cells may benefit from a *Candida* vaccine. Vaccination can augment specific host defenses to compensate for a suboptimal or partially defective anti-*Candida* host defense. However, vaccines may be less effective for severely immunocompromised patients at risk for candidiasis due to compromised antigen presentation as well as a B and T cell–driven immunity. Finally, it is difficult to predict how increasing host immunity to *Candida* through vaccination would impact diseases associated with strong *Candida-*specific immunity. Vaccination may augment the *Candida-*specific T_H_17 and antibody-mediated immune responses that, as discussed above, can play a role in pulmonary inflammatory pathology and IBDs.

Given the potential beneficial role of *C*. *albicans* commensalism, vaccination may negatively impact these beneficial effects by compromising intestinal colonization. A viable strategy circumventing commensalism may be the neutralization of specific virulence factors by passive immunization. Since the currently developed vaccines specifically target hyphal proteins, they may not necessarily impact *C*. *albicans* colonization. In fact, by specifically targeting the filamentous infectious morphology, these vaccines may even reinforce commensalism. However, at this stage, we do not understand which fungal factors or activities trigger the beneficial effects, and the hyphal morphology may also play a role during commensalism, and, thus, effects of hyphae-specific vaccination on colonization remain to be elucidated. Other vaccine designs such as a multivalent vaccine against *C*. *albicans* or immunization with inactivated yeast cells may also induce potent responses against yeast cells and thereby compromise the commensal state. Nevertheless, with currently only one vaccine in clinical trials for vulvovaginal candidiasis [22], it is unlikely that we will obtain insights into the effect of anti-*Candida* vaccination on intestinal commensalism and human health in the near future. Furthermore, it remains to be investigated which mechanisms cause the detrimental effects associated with *C*. *albicans* gut colonization and immunopathology at other body sites.

## Conclusions

Given the parallel evolutionary history of *C*. *albicans* and humans and the fact that a potential harmful fungus would be subjected to negative selection, it was predicted that intestinal colonization with this yeast may have beneficial effects. It has become clear that colonization triggers a set of immunological mechanisms that either contribute to diseases such as IBDs and asthma or drive protective innate and adaptive immune responses, largely depending on the extent, timing, and nature of the response. Many studies use mice as a model organism, yet caution must be taken in their interpretation since *C*. *albicans* is not a normal commensal of many laboratory mice and important differences between the murine and human immune system exist. However, a key message is that *C*. *albicans* can elicit protective immunity against *C*. *albicans* infections, but also other fungi and bacteria. The studies discussed here suggest that *C*. *albicans* should be considered as a therapeutic target in the treatment of IBDs and to prevent *C*. *albicans* infections, but also as a potent vehicle to promote immunization against other pathogens.
